# Chromodomain Helicase DNA Binding Protein 1-like, a negative regulator of Forkhead box O3a, promotes the proliferation and migration of Angiotensin II-induced vascular smooth muscle cells

**DOI:** 10.1080/21655979.2021.2019869

**Published:** 2022-01-09

**Authors:** Xueyao Zhang, Yingxian Sun

**Affiliations:** Department of Cardiovascular Medicine, The First Hospital of China Medical University, Shenyang, People’s Republic of China

**Keywords:** Chromodomain Helicase DNA-Binding Protein 1-Like, essential hypertension, Forkhead box O3a, vascular smooth muscle cells

## Abstract

Essential hypertension (EH) represents a major risk factor for stroke, myocardial infarction, and heart failure. Dysregulated proliferation and migration of vascular smooth muscle cells (VSMCs) play an important role in pathogenesis of EH. This study aims to investigate the effect of Chromodomain Helicase DNA Binding Protein 1-Like (CHD1L) on Angiotensin II (AngII)-induced VSMCs injury and reveal the underlying mechanism. The expression of CHD1L in EH patients was determined by bioinformatics analysis, and then it was silenced in AngII-induced VSMCs to detect the changes in cellular functions including proliferation, migration, invasion and phenotypic switching via CCK-8, EDU staining, wound healing, transwell and Western blot assays, respectively. Inflammation and oxidative stress were also measured by detecting related markers via commercial kits. After confirming the binding sites between forkhead box O3A (FOXO3a) and CHD1L and their negative association by bioinformatics analysis, FOXO3a was further silenced, and the cellular functions were assessed again to reveal the underlying mechanism. Results showed that CHD1L was highly expressed in EH, and interference of CHD1L suppressed the proliferation, migration, invasion and phenotypic switching in VSMCs. Inflammation and oxidative stress were also restrained by CHD1L knockdown. After validating the negative role of FOXO3a in regulating CHD1L, it was found that FOXO3a abrogated the effect of CHD1L knockdown on the cellular functions of AngII-induced VSMCs. In conclusion, FOXO3a suppresses the proliferation and migration of AngII-induced VSMCs by down-regulating CHD1L.

## Introduction

As a multifactorial disease affected by the interplay of environmental and genetic factors, essential hypertension (EH) is threatening the health of some 40% of people in the world, constituting a major health problem [1,2]. According to recent statistics, there are 10.4 million deaths from EH worldwide every year [3]. Moreover, it is such a disease with an incredibly increasing incidence that a large majority of people over 25 are believed to suffer from EH [4]. The molecular dynamics underlying EH pathogenesis remains not understood, leading to slow progression in the therapies for EH [5,6]. Thus, clarifying the molecular mechanism of EH is of great urgency for the acceleration of related treatment methods for clinical use.

Vascular smooth muscle cells (VSMCs) constitute the bulk of the vascular wall and are closely involved in the processes of vascular changes including endothelial dysfunction, increased vascular contraction, and arterial remodeling, thus serving as a common cause of vascular proliferative diseases, such as EH, atherosclerosis and restenosis after balloon angioplasty [7,8]. Upon vascular injury, such as renin-angiotensin-aldosterone system, oxidative stress, inflammation and vascular changes, VSMCs can dedifferentiate from a ‘contractile’ phenotype to a highly ‘synthetic’ phenotype, characterized by an enhanced rate of proliferation, migration, and reduction of VSMC-specific markers, which may contribute to the development of atherosclerosis, hypertension, and neointima formation [9]. Therefore, Angiotensin II (AngII)-stimulated VSMCs were widely used to simulate the pathological conditions occurring in EH [10,11].

After analyzing the data from Gene Expression Omnibus (GEO) datasets GSE24752 database, it was found that the expression of Chromodomain Helicase DNA Binding Protein 1 Like (CHD1L) in the peripheral blood of patients with EH was higher than that in volunteers with normal blood pressure. Human CHD1L gene, also known as ALC1, was identified by Ma et al. in 2008 [12]. Afterward, CHD1L was identified as an oncogene and was found to exhibit an oncogenic role during malignant transformation of multiple cancers [13–15]. The promotive effect of CHD1L on cancer cells proliferation and migration has also been reported [16–18]. However, whether CHD1L plays a role in EH via regulating VSMCs proliferation and migration has not been studied.

By analyzing the JASPAR database, we found that a member of the forkhead box class O (FOXO) family, FOXO3 (also known as FOXO3a), could bind to the promoter of CHD1L. Notably, FOXO3a has been indicated to play an important role in hypertension and functions of VSMCs [19–21].

Therefore, we speculated that, upon binding to FOXO3a, CHD1L may play a role in EH via regulating VSMCs proliferation and migration. This study aimed to investigate the effect of CHD1L/FOXO3a axis on AngII-induced VSMCs injury.

## Materials and methods

### Bioinformatics analyses

The sequence analysis on the peripheral blood of volunteers with normal blood pressure and that of EH patients was conducted in GEO datasets GSE24752 (https://www.ncbi.nlm.nih.gov/geo/query/acc.cgi?acc=GSE24752) database [22] using GEO2R. The interaction of FOXO3 with CHD1L promoter was examined by JASPAR (http://jaspar.genereg.net) [23].

### Cell culture and transfection

The human VSMCs (ATCC) were maintained in DMEM with 10% fetal bovine serum (FBS, Hyclone, USA), penicillin (100 U/ml) and streptomycin (100 g/ml) in a humidified incubator with 5% CO_2_ at 37°C [24]. For AngII (MedChemExpress, China) treatment, VSMCs were exposed to different concentrations of AngII for different hours.

To knockdown or overexpress CHD1L or FOXO3a in VSMCs, the small interfering RNAs (siRNAs) against FOXO3a and CHD1L and their respective control siRNAs (negative control), and the plasmid overexpressing FOXO3a and its control (Ov-NC) were purchased from Ribo Biotech Co., Ltd. (Guangzhou, Guangdong, China). To eliminate the off-target effects, two siRNAs (si-CHD1L/FOXO3a#1 and #2) were constructed, and cells (2 × 10^5^ cells/well) were transfected using Lipofectamine 2000 (Invitrogen) according to the manufacturer’s protocol as previously reported [25]. At 48 h post-transfection, transfection efficiency was measured, and the siRNAs with better transfection efficiency were used for further functional analysis.

### Real-time quantitative PCR (RT-qPCR)

To assess the mRNA level of genes in VSMCs, RT-qPCR was performed. Total RNA was extracted from VSMCs with TRIzol Reagent (Invitrogen) according to the manufacturer’s instructions. The synthesis of total RNA into cDNA was performed by All-in-One First-Strand cDNA Synthesis Kit (Tiangen, Beijing, China) following the manufacturer’s protocol. The amplification conditions were 95°C for 30 sec, 55°C for 1 min, and 72°C for 2 min for 45 cycles. The primer sequences were as follows: FOXO3a, 5ʹ-GGAACGUGAUGCUUCGCAATT-3ʹ (sense) and 5ʹ- GTCTTCAGGTCCTCCTGTTCCT-3ʹ (anti-sense); CHD1L, 5ʹ- CACCAAAGAAGAGAGAAGGAGAGT-3ʹ (sense) and 5ʹ- GGCTCTTTCCTCCTTGTCGC-3ʹ (anti-sense); GAPDH 5ʹ-AATGGGCAGCCGTTAGGAAA-3ʹ (sense), reverse 5ʹ-GCGCCCAATACGACCAAATC-3ʹ (anti-sense). Appl-ied Biosystems 7900 Real-Time PCR System (Applied Biosystems, Foster City, CA) was used for RNA quantification assay, and GAPDH was used as the internal control. Fold change was determined by the 2^−ΔΔCt^ method [26].

### Western blot

Western blot was performed as previously reported [27] to measure the expression level of proteins in VSMCs. Protein was extracted from the VSMCs, homogenized and lysed in RIPA Lysis Buffer (Beyotime) at 4°C. Protein concentrations were quantified by the Bicinchoninic Acid (BCA) Protein Assay Kit (BioRad, Hercules, CA, USA). Equal amounts of proteins were separated by SDS-PAGE for electrophoresis and transferred onto polyvinylidene difluoride (PVDF) membranes (Millipore, USA). After being blocked in 5% skim milk at room temperature for 2 h, primary antibodies were used to incubate the membranes overnight at 4°C. GAPDH was considered as an internal reference. After the PVDF membranes were incubated with the secondary antibody (1:10,000 dilution, Cell Signaling Technology, USA) at room temperature for 2 h, protein bands were developed using chemiluminescence, and band intensity was analyzed using image J analysis software.

### Cell counting kit-8 (CCK-8)

CCK-8 assay was performed as previously reported [28] using the CCK-8 Kit (Beyotime, China). VSMCs were seeded into a 96-well plate at a density of 2 × 10^4^ cells/well. 48 h after transfection, VSMCs were challenged with indicated concentrations of AngII for indicated hours, then 10 μl of CCK8 solution was added into the cells for 2 h. The absorbance at 450 nm was measured with a microplate reader (ELX800; USA) with optical density at 450 nm..

### *EDU* (*5-ethynyl-2ʹ-deoxyuridine)*

EDU staining was used to detect cell proliferation [29]. VSMCs were cultured into 96-well plates at a density of 2 × 10^4^ cells/well and incubated for 48 h, and then a new medium containing 100 μL of 50 μM EdU (Abcam) substituted the original medium. After another 2 h of incubation later, cells were fixed with 4% formaldehyde for 30 min and treated with 0.5% Triton X-100 for 20 min. After washing with PBS for twice, cells were incubated with EDU Reaction Mix containing iFluor 488 azide (Abcam) for 30 min, and then counterstained with DAPI for nucleus staining for 30 min. Then, cells were visualized by an inverted fluorescence microscope (Leica) with Ex/Em = 491/520 nm

### Cell migration assay

Cell migration was assessed by a wound-healing assay as previously reported [25]. Briefly, the VSMCs (3 × 10^5^ cells/well) were seeded in 6-well plates and allowed to grow to confluence. The wound was created on a monolayer of the cells with a standard 1-ml pipette tip. The cells remaining on the upper membrane were removed by a cotton swab, and the medium was replaced with a serum-free medium. After culture for 48 h, images of the wound area were captured at 0 and 48 h under a microscope (magnification x200; Leica DM4000, Buffalo Grove, USA).

### Cell invasion assay

Cell invasion was evaluated using the transwell chamber (Corning, USA) as previously reported [25]. 2 × 10^4^ VSMCs were seeded into the upper chamber, which was previously coated with 300 μL serum-free medium. The lower chamber of the plate was supplemented with 500 μL medium with 10% FBS. After incubation for 48 h, the cells that invaded into the lower-chamber were fixed with methanol and stained with 0.1% crystal violet. Invaded cells were counted and captured under a microscope (magnification x200; Leica DM4000, Buffalo Grove, USA).

### Detection of inflammation and oxidative stress

The levels of the pro-inflammatory cytokines TNF-α (cat. no. ab181421), IL-1β (cat. no. ab214052) and IL-6 (cat. no. ab178013) were detected in VSMCs using their respective ELISA kits (Abcam) as per the manufacturer’s recommendations as previously reported [30]. The NADPH oxidase activity and reactive oxygen species (ROS) level were detected by the NADP+/NADPH Assay Kit (cat. no. S0179; Beyotime) ROS Assay Kit (cat. no. S0033M; Beyotime), respectively, according to the manufactures’ protocol.

### Dual luciferase reporter assay

The binding between CHD1L and FOXO3a was determined by dual-luciferase reporter assay as previously reported [27]. Briefly, the human VSMCs were inoculated into 96-well plates at a density of 2 × 10^4^ cells per well. When the cell confluence reached 60%, the Renilla luciferase reporter pRL-TK plasmids containing wild-type or mutant 3ʹ-untranslated regions (UTRs) of CHD1L (Promega Corporation) were transfected into cells alongside Ov-FOXO3a using Lipofectamine 2000 at 37°C. After incubation at 37°C for 48 h, luciferase intensity was examined using a Dual-Luciferase assay kit (Promega Corporation) by comparing firefly with Renilla luciferase activity.

### *Chromatin immunoprecipitation (CHIP*)

The binding between CHD1L promoter and FOXO3a was determined by CHIP assay as previously reported [31]. The CHIP assay was performed using a CHIP assay kit (Thermo Fisher Scientific, Inc.). Briefly, the cells were fixed by 1% formaldehyde for 10 min and stopped by 125 mM glycine. The cells were then lysed in a lysis buffer and added with protease inhibitors. Then, the chromatin fragments were immunoprecipitated with anti-FOXO3a or IgG (Santa Cruz Biotechnology) at 4°C overnight. All antibodies were pre-mixed with the magnetic beads in kit (Thermo Fisher Scientific, Inc.) and incubated for 1 h at 4°C. On the next day, immunoprecipitated products of genomic DNA were treated with the CHIP elution buffer (1% SDS, 100 mM NaCO_3_) and centrifuged at 6000xg for 1 min. Subsequently, precipitated DNA samples were detected using RT-qPCR for analyzing CHD1L mRNA level.

### Statistical analysis

The data were expressed as the mean ± standard deviation (SD) from three independent experiments. The data from different groups were compared by one-way analysis of variance (ANOVA), followed by Tukey’s test. Statistical analyses were performed using GraphPad Prism 6.0 (GraphPad Software Inc.), and p < 0.05 was considered statistically significant.

## Results

### CHD1L is upregulated in EH and AngII-induced VSMCs

**Figure 1.**
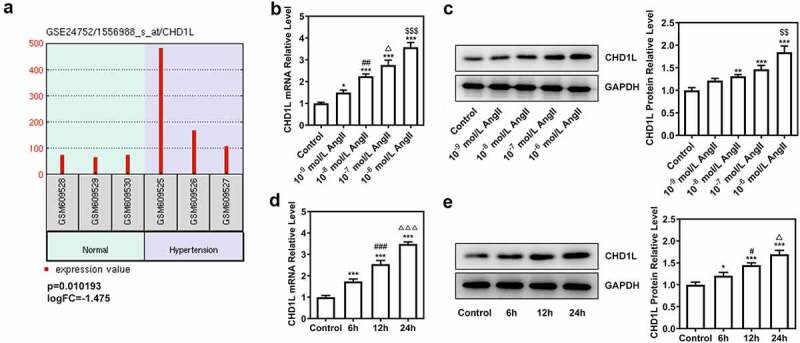
CHD1L is upregulated in EH and AngII-induced VSMCs. (a) The expression analysis of CHD1L in the peripheral blood of volunteers with normal blood pressure and EH patients using the data from Gene Expression Omnibus (GEO) datasets GSE24752. (b-c) VSMCs were exposed to different concentrations of AngII for 24 h, then the mRNA and protein expression of CHD1L in VSMCs were detected by RT-qPCR and Western blot assays, respectively. *P < 0.05, **P < 0.01 and ***P < 0.001 vs Control; ^##^P < 0.01 vs 10^−9^ mol/L AngII; ^Δ^P<0.05 vs 10^−8^ mol/L AngII; ^$$^P < 0.01 and ^$$$^P < 0.001 vs 10^−7^ mol/L AngII. (d-e) VSMCs were exposed to 10^−6^ mol/L AngII for different hours, then the mRNA and protein expression of CHD1L in VSMCs were detected by RT-qPCR and Western blot assays, respectively. *P < 0.05 and ***P < 0.001 vs Control; ^#^P < 0.05 and ^###^P < 0.001 vs 6 h; ^Δ^P<0.05 and ^ΔΔΔ^P<0.001 vs 12 h. CHD1L, Chromodomain Helicase DNA Binding Protein 1-Like; EH, essential hypertension; VSMCs, vascular smooth muscle cells; AngII, Angiotensin II.

Firstly, to determine whether CHD1L plays a role in EH, we analyzed the GSE24752 database and found that the expression analysis on CHD1L in the sequencing of the peripheral blood in volunteers with normal blood pressure and that in EH patients showed high expression of CHD1L in the peripheral blood of EH patients (p = 0.010193, logFC = −1.475) compared with normal control (Figure 1(a)). Following that, AngII at different concentrations was used to induce the VSMCs for 24 h and at 10^−6^ mol/L was used to treat VSMCs for different time points. Results showed that CHD1L expression in ASMCs was significantly increased upon AngII treatment in concentration- and time-dependent manners. As AngII at a concentration of 10^−6^ mol/L for 24 h of incubation achieved the best effects on elevating the expression of CHD1L, we chose it for the subsequent experiments (Figure 1(b-e)).

### CHD1L deficiency suppresses the proliferation, migration, phenotypic switching and inflammation of AngII-induced VSMCs

**Figure 2.**
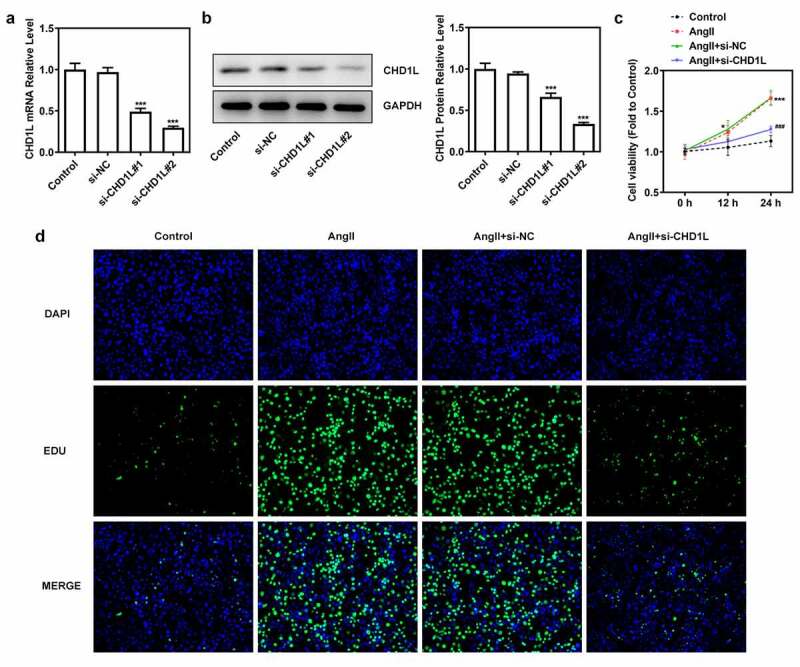
CHD1L deficiency suppresses the proliferation of AngII-induced VSMCs. (a-b) VSMCs were transfected with indicated siRNAs or not, then the transfection efficiency was confirmed by RT-qPCR and Western blot assays, respectively. ***P < 0.001 vs si-NC. (c) Control or transfected VSMCs were treated with 10^−6^ mol/L AngII, at 0, 12 and 24 h post-treatment, the cell viability was measured by CCK-8 assay. *P < 0.05 and ***P < 0.001 vs Control; ^###^P < 0.001 vs AngII + si-NC. (d) Control VSMCs or VSMCs that transfected with si-NC or si-CHD1L were treated with 10^−6^ mol/L AngII for 24 h, then cell proliferation were measured by EDU staining (magnification x200). CHD1L, Chromodomain Helicase DNA Binding Protein 1-Like; VSMCs, vascular smooth muscle cells; AngII, Angiotensin II; si, small interfering RNA; NC, negative control.

**Figure 3.**
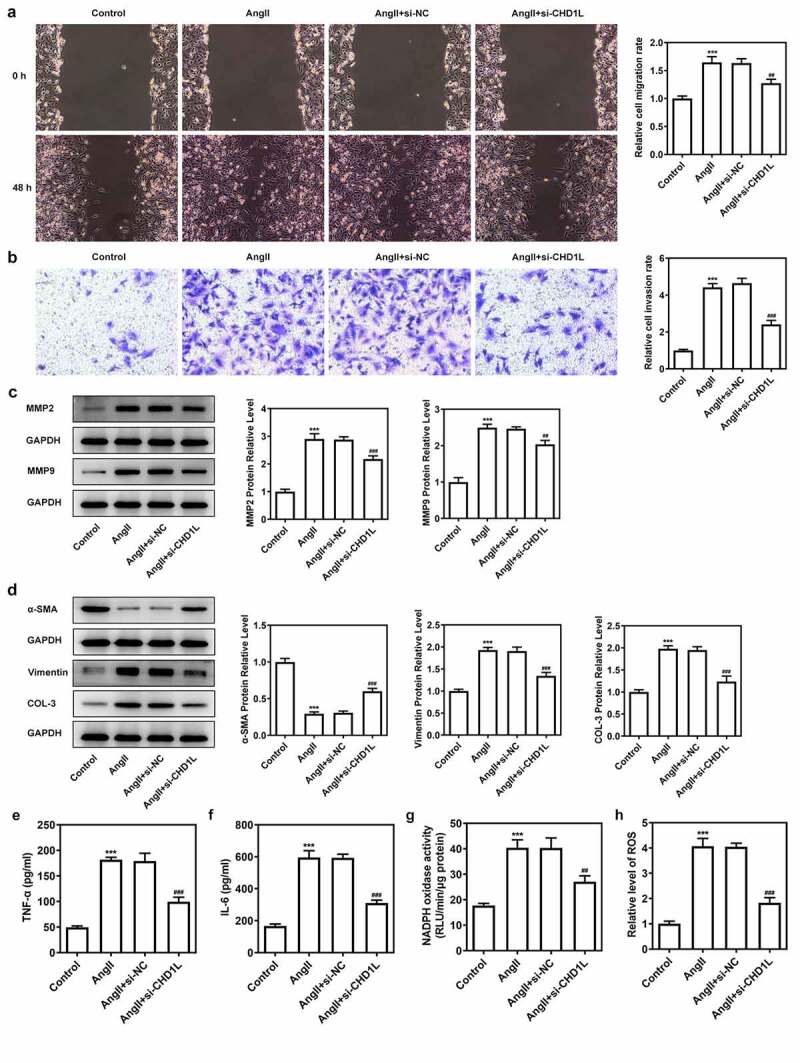
CHD1L deficiency suppresses the migration, phenotypic switching and inflammation of AngII-induced VSMCs. The control VSMCs or VSMCs that transfected with si-NC or si-CHD1L were treated with 10^−6^ mol/L AngII for 24 h, then (a) migration was measured by wound healing assay (magnification x200); (b) invasion was detected by transwell assay (magnification x200); (c) migration- and invasion-related proteins and (d) phenotypic switching-related proteins expression levels were assessed by Western blot; (e-f) the production of pro-inflammatory cytokines including TNF-α and IL-6 was measured by ELISA kits; (g-h) the activity of NADPH oxidase and the level of ROS were detected by corresponding kits. ***P < 0.001 vs Control; ^##^P < 0.01 and ^###^P < 0.001 vs AngII + si-NC. CHD1L, Chromodomain Helicase DNA Binding Protein 1-Like; VSMCs, vascular smooth muscle cells; AngII, Angiotensin II; si, small interfering RNA; NC, negative control; MMP, matrix metalloproteinase; COL-3, collagen-3.

To investigate the molecular mechanism of CHD1L in EH, the cellular functions including invasion, migration and phenotypic switching of AngII-induced VSMCs were further explored. We silenced CHD1L and si-CHD1L#2, which showed lower expression than si-CHD1L#1, was selected for further experiments (Figure 2(a,b)). As exhibited in (Figure 2(c,d)), CHD1L deficiency suppresses the cell viability and excessive proliferation of AngII-induced VSMCs. Obviously, AngII remarkably increased the invasive and migration rates of VSMCs, whereas genetic inhibition of CHD1L greatly abolished this situation (Figure 3(a,b)). Similar results were observed in (Figure 3(c)), which showed that AngII significantly increased the expression of matrix metalloproteinase (MMP) 2 and MMP9, while CHD1L silence reversed this effect. Compelling evidence has implied that phenotypic switching of VSMCs plays significant effects on EH [32], and thus the expression changes of proteins related to phenotypic switching were measured. Upon AngII challenge, VSMCs were changed to a synthetic phenotype, as evidenced by the decreased alpha-smooth muscle actin (α-SMA) and elevated Vimentin and collagen-3 (COL-3) expression (Figure 3(d)). However, this trend was reversed after the expression of CHD1L was inhibited in AngII-induced VSMCs. Overall, CHD1L deficiency suppresses the invasion, migration and phenotypic switching of AngII-induced VSMCs.

Next, the inflammation and oxidative stress of AngII-induced VSMCs transfected with si-CHD1L were measured. Results in (Figure 3(e–h)) demonstrated that the levels of proinflammatory factors TNF-α and IL-6 were enhanced by AngII while inhibition of CHD1L reduced their levels. Meanwhile, AngII induced high level of NADPH oxidase activity and ROS production, which was reversed when CHD1L was silenced. Taken together, these results demonstrate that CHD1L deficiency suppresses the inflammation and oxidative stress of AngII-induced VSMCs.

### FOXO3a negatively regulates CHD1L

**Figure 4.**
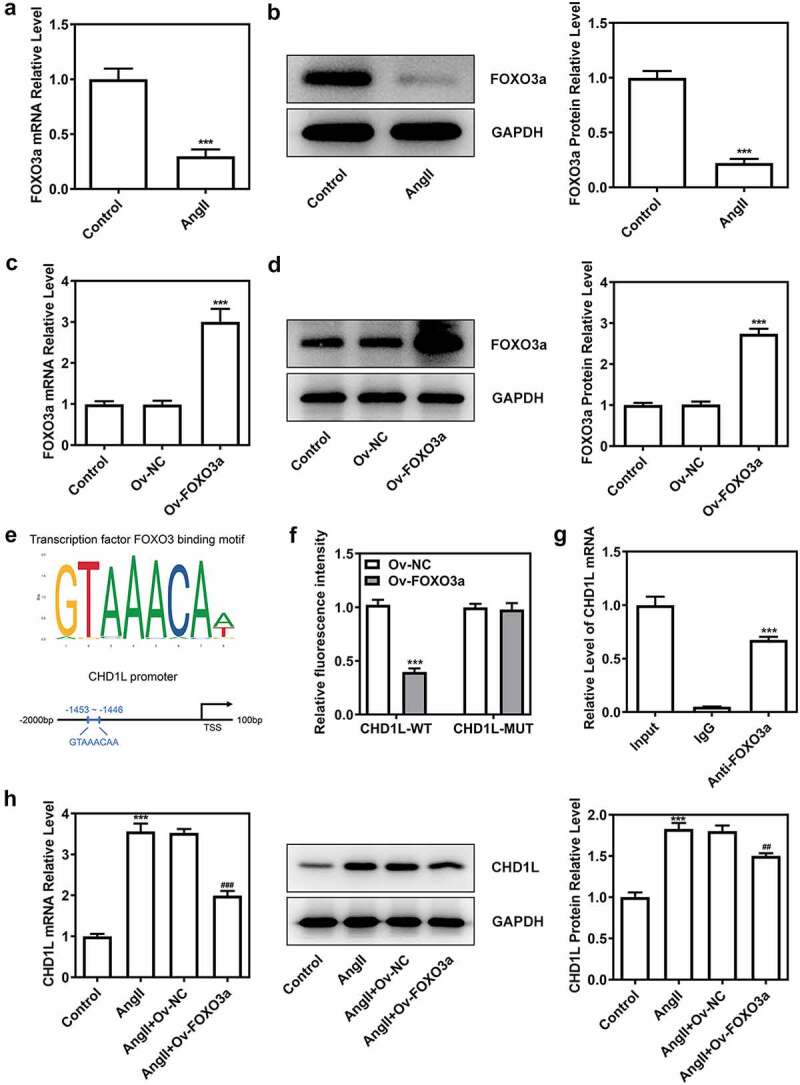
FOXO3a negatively regulates CHD1L in VSMCs. (a-b) VSMCs were treated with 10^−6^ mol/L AngII for 24 h or not, then the expression of FOXO3a was confirmed by RT-qPCR and Western blot assays. ***P < 0.001 vs Control. (c-d) The transfection efficiency was confirmed by RT-qPCR and Western blot assays after FOXO3a was overexpressed in VSMCs. ***P < 0.001 vs Ov-NC. (e) The binding sites between FOXO3a and CHD1L promoter were predicted by JASPAR database. (f) The promoter region activity and the (g) association between FOXO3a and CHD1L were measured by dual luciferase reporter assay and CHIP assay, respectively. ***P < 0.001 vs CHD1L-WT + Ov-NC and ***P < 0.001 vs IgG, respectively. (h) The expression of CHD1L in control or AngII-induced VSMCs transfected with Ov-FOXO3a or Ov-NC was measured by RT-qPCR and Western blot assays. ***P < 0.001 vs Control; ^##^P < 0.01 and ^###^P < 0.001 vs AngII + Ov-NC. CHD1L, Chromodomain Helicase DNA Binding Protein 1-Like; FOXO3a, Forkhead box O3a; VSMCs, vascular smooth muscle cells; AngII, Angiotensin II; Ov, overexpression; NC, negative control. CHIP, chromatin immunoprecipitation.

To determine the function of FOXO3a as a transcriptional regulator, the JASPAR database was used, and the binding sites between FOXO3a and CHD1L were predicted (Figure 4(e)). Then, we found that the expression of FOXO3a was reduced in AngII-induced VSMCs, in contrast with the control group (Figure 4(b)). To obtain further information about the relationship between FOXO3a and CHD1L, FOXO3a was overexpressed in VSMCs (Figure 4(c,d)). Dual-luciferase reporter assay showed that FOXO3a promoter activity was rescued when the VSMCs were transfected with the mutant promoter construct (Figure 4(f)). Further, ChIP assay validated the binding of FOXO3a to CHD1L (Figure 4(g)). The overexpression of FOXO3a rescued the expression of CHD1L promoted by AngII, suggesting that FOXO3a negatively regulated CHD1L (Figure 4(h)).

### Downregulation of FOXO3a reverses the effect of CHD1L deficiency on AngII-induced VSMCs

**Figure 5.**
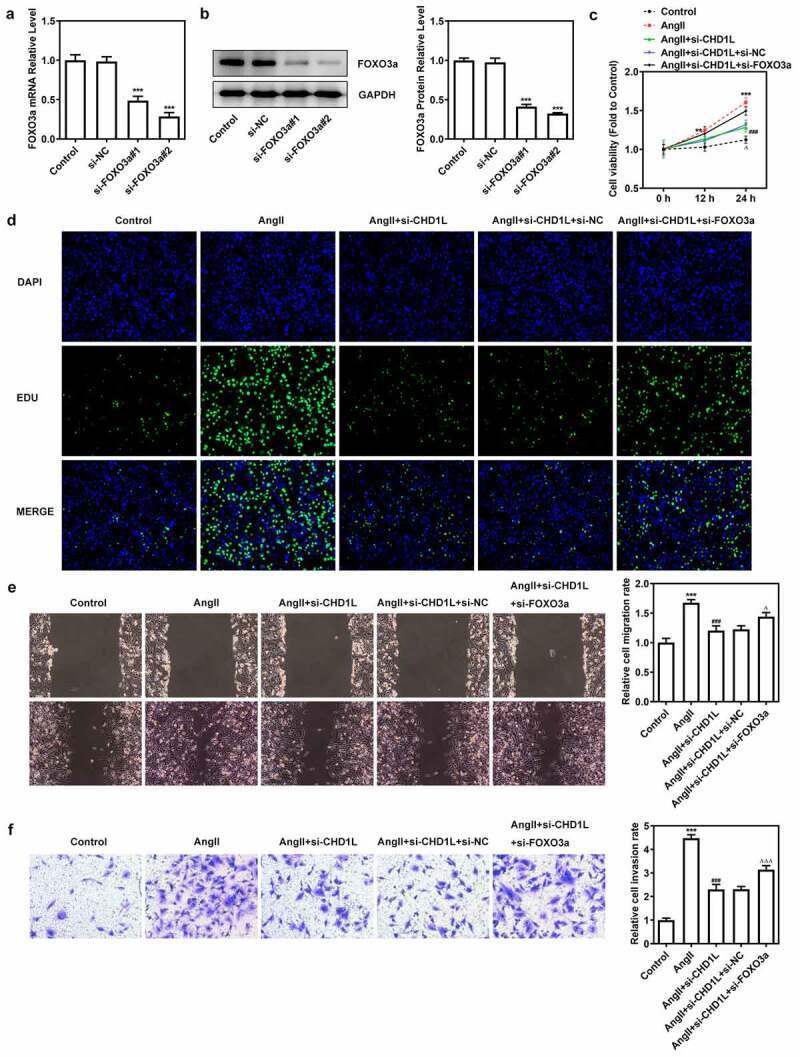
Downregulation of FOXO3a reverses the effect of CHD1L deficiency on AngII-induced VSMCs. (a-b) The transfection efficiency was confirmed by RT-qPCR and Western blot assays after FOXO3a was silenced in VSMCs. ***P < 0.001 vs si-NC. (c) Control or transfected VSMCs were treated with 10^−6^ mol/L AngII, at 0, 12 and 24 h post-treatment, the cell viability was measured by CCK-8 assay. (d-f) The control VSMCs or VSMCs that transfected with indicated siRNAs were treated with 10^−6^ mol/L AngII for 24 h, then (d) cell proliferation was observed by EDU staining (magnification x200); (e) migration was measured by wound healing assay (magnification x200); (f) invasion was detected by transwell assay (magnification x200). **P < 0.01 and ***P < 0.001 vs Control; ^###^P < 0.001 vs AngII; ^Δ^ P < 0.05 and ^ΔΔΔ^P<0.001 vs AngII + si-CHD1L+ si-NC. CHD1L, Chromodomain Helicase DNA Binding Protein 1-Like; FOXO3a, Forkhead box O3a; VSMCs, vascular smooth muscle cells; AngII, Angiotensin II; si, small interfering RNA; NC, negative control.

**Figure 6.**
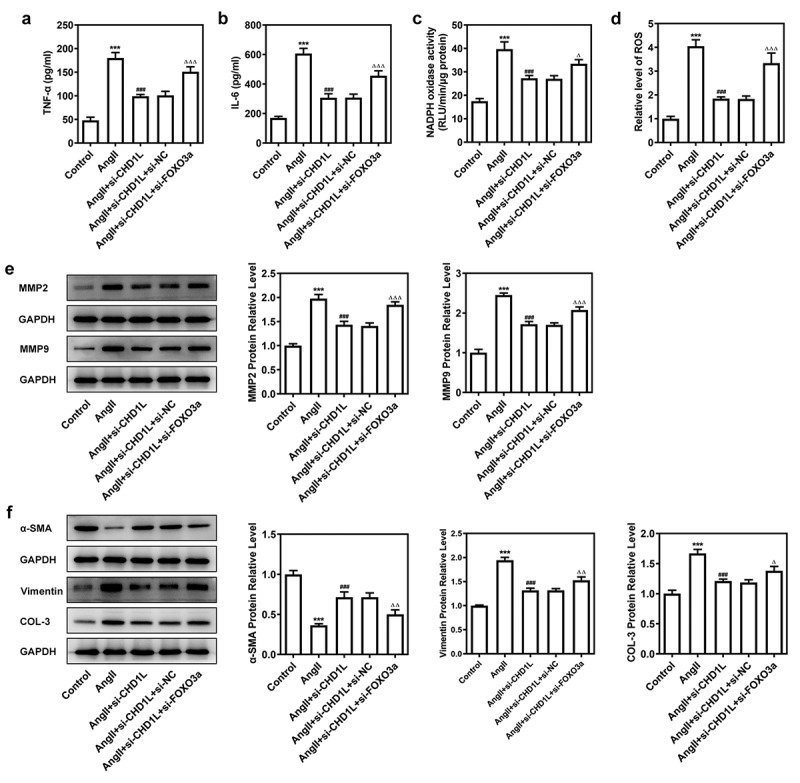
Downregulation of FOXO3a reverses the effect of CHD1L deficiency on AngII-induced VSMCs. The control VSMCs or VSMCs that transfected with indicated siRNAs were treated with 10^−6^ mol/L AngII for 24 h, then (a-b) the production of pro-inflammatory cytokines including TNF-α and IL-6 was measured by ELISA kits; (c-d) the activity of NADPH oxidase and the level of ROS were detected by corresponding kits; the expression of (e) migration- and invasion-related proteins and (f) phenotypic switching-related proteins was assessed by Western blot. ***P < 0.001 vs Control; ^###^P < 0.001 vs AngII; ^Δ^P<0.05, ^ΔΔ^P<0.01 and ^ΔΔΔ^P<0.001 vs AngII + si-CHD1L+ si-NC. CHD1L, Chromodomain Helicase DNA Binding Protein 1-Like; FOXO3a, Forkhead box O3a; VSMCs, vascular smooth muscle cells; AngII, Angiotensin II; si, small interfering RNA; NC, negative control; MMP, matrix metalloproteinase; COL-3, collagen-3.

We then explored the consequences of FOXO3a knockdown in AngII-induced VSMCs with CHD1L deficiency. After knocking down FOXO3a, si-FOXO3a#2 that exhibited lower expression than si-FOXO3a#1 was chosen for subsequent experiments (Figure 5(a,b)). As depicted in (Figure 5(c,d)), the cell viability and proliferation of AngII-induced VSMCs inhibited by CHD1L deficiency were rescued by si-FOXO3a. Furthermore, regarding migration and invasion, AngII greatly prompted the migration and invasive rates of VSMCs, whereas CHD1L deficiency reduced and si-FOXO3a promoted their rates (Figure 5(e,f)). si-FOXO3a also abrogated the inhibitory effects of CHD1L deficiency on inflammation and oxidative stress of AngII-induced VSMCs (Figure 6(a–d)). Consistent results could be found in (Figure 6(e,f)). Thus, downregulation of FOXO3a reverses the effect of CHD1L deficiency on AngII-induced VSMCs.

## Discussion

VSMCs, which constitute the majority of the arterial wall, are accountable for maintaining vascular tone in response to hemodynamic changes and humoral stimulation [33]. The dysfunction of AngII-induced VSMCs, such as increased oxidative stress, inflammation, migration, and hyperplasia, plays an essential role in the pathogenesis of hypertension [34–36]. In this study, we found that silence of CHD1L in VSMCs significantly inhibited the AngII-induced injuries in VSMCs. In addition, we demonstrated that the negative regulation of FOXO3a on CHD1L was responsible for the effect of CHD1L on AngII-induced VSMCs.

Since the role of CHD1L was reported to be limited to the field of tumors, we innovatively investigated the function of CHD1L in EH. In this study, we first assessed the expression of CHD1L in the peripheral blood of EH patients and people with normal blood pressure, observing the abnormally high expression of CHD1L. After AngII induction, the expression of CHD1L was also elevated, indicating the potential of CHD1L as a diagnostic marker of EH. We also reported reduced proliferation, migration, and invasion rate, as well as protein expression of MMP2 and MMP9 in AngII-induced VSMCs in the presence of CHD1L siRNA. Increased MMP2 and MMP9 expressionS have been indicated to promote VSMCs migration [37], implying that the occurrence of excessive VSMCs proliferation, migration and invasion as an outcome of AngII stimulation might be rescued by CHD1L interference.

Upon the stimulation of various inducements, VSMCs will undergo a phenotypic transformation from a differentiated phenotype to a dedifferentiated phenotype, involving reduced expression of contractile proteins, and increased release of extracellular matrix and proinflammatory factors [38,39]. Concurrently, AngII induced downregulated level of α-SMA and upregulated levels of Vimentin and COL-3 in VSMCs, which were abrogated in the presence of CHD1L. α-SMA is a VSMC marker protein, while Vimentin and COL-3 are the phenotypic switching markers [40]. These results indicated the involvement of CHD1L in prompting the phenotypic switching of VSMCs, thereby manipulating the progression of EH. Chronic inflammation and oxidative stress of VSMCs is an essential pathophysiological process in the initiation and progression of cardiovascular diseases, such as hypertension [41,42]. Increased NADPH oxidase and ROS level can aggravate atherosclerosis and hypertension [43]. The expression changes in inflammation- and oxidative stress-related markers indicated that interference of CHD1L also suppressed the inflammation and oxidative stress of AngII-induced VSMCs.

FOXO3a has been shown to have a wide range of transcriptional targets in a large number of physiological processes [44]. FOXO3a is capable of modulating blood pressure in pregnant hypertension rats as a downstream target of miR-155 [21]. The activation of FOXO3a by SGK1 led to fluid retention and hypertension, which would further induce the occurrence of type 2 diabetes [45]. Based on the above pieces of evidence, it would be better to target FOXO3a, which may play a significant role in modulating blood pressure. Importantly, in the present study, we identified that FOXO3a could bind to the promoter region of CHD1L. In AngII-induced VSMCs, FOXO3a overexpression downregulated the expression of CHD1L. We, therefore, speculated that FOXO3a may play its role in EH via targeting CHD1L and negatively regulating CHD1L expression. In an earlier study, FOXO3a activity is increased in VSMCs in human atherosclerotic plaques [46,47]. In the present study, similar results were obtained, which showed that FOXO3a was markedly up-regulated in AngII-induced VSMCs, indicating its potential role in AngII-induced VSMCs injury. Subsequently, we found that si-FOXO3a reversed all the inhibitory effects of CHD1L knockdown on the cellular activities including proliferation, migration, invasion, phenotypic switching, inflammation and oxidative stress stimulated by AngII.

## Conclusion

In summary, we elucidate several novel findings, including the dysregulated expression of CHD1L in EH, the stimulative impacts of CHD1L on promoting proliferation, inflammation, oxidative stress and phenotypic switching of VSMCs, and recognition of FOXO3a as its negative transcriptional regulator. The conclusion that FOXO3a participates in the proliferation and migration of AngII-induced VSMCs by regulating CHD1L can be drawn. Nevertheless, further investigation into the specific regulatory role between CHD1L and FOXO3a in EH along with the molecular mechanism of CHD1L/FOXO3a in EH in vivo should be conducted for the clinical application of targeted therapies against EH.

## Highlights

- CHD1L is upregulated in hypertension.
- CHD1L deficiency suppresses the proliferation, migration, phenotypic switching and inflammation of AngII-induced VSMCs
- FOXO3a negatively regulated CHD1L expression to play a role in hypertension.
